# P10 Development of a database to enable real time efficient recording of antimicrobial ward round stewardship interventions and support ongoing measurement of antimicrobial stewardship improvement

**DOI:** 10.1093/jacamr/dlae217.014

**Published:** 2025-01-23

**Authors:** Rachael Rodger, Fiona Kinnaird, Beth White, Karen Downie

**Affiliations:** NHS Greater Glasgow & Clyde, Glasgow, Scotland; NHS Greater Glasgow & Clyde, Glasgow, Scotland; NHS Greater Glasgow & Clyde, Glasgow, Scotland; NHS Greater Glasgow & Clyde, Glasgow, Scotland

## Abstract

**Background:**

Antimicrobial stewardship (AS) initiatives are crucial in the fight against antimicrobial resistance (AMR).^1^ Using quality improvement (QI) methodology we can adapt our AS initiatives to meet the demands of the complex healthcare environment.^2^ To determine if our AS initiatives are effective, we need to measure if improvement has occurred, but this can be time consuming and challenging in the acute hospital setting. Antimicrobial consumption is often used as an indicator measure of AS, however, using technology we can develop more reliable and specific measures of AS and patient-centred care improvement.^3^ An infectious diseases (ID) physician and antimicrobial pharmacist (AMP) ward round (AWR) service was recently introduced to the Royal Alexandra Hospital. To enable measurement of the impact of this service on AS within the hospital it was decided to develop an antimicrobial ward round (AWR) database.

**Objectives:**

To develop a database to enable real time efficient data collection of AWR outcomes and AS interventions to enable more accurate measurement of AS improvement.

**Methods:**

The AMP, data analyst and ID physician worked collectively to co-design the format of the database using an Access^®^ system. Outcome definitions^4^ and AS intervention definitions were agreed and standardized. The database was piloted over a two month period with continuous feedback and adjustments to improve ease and efficiency of data input. The updated database was then used to collect outcome and AS interventions from over 200 AWR consultations. This data was compiled and used to measure AS improvement following introduction of the AWR service.

**Results:**

The database enabled efficient real time data collection and a more accurate and standardized approach to AS data collection and report generation. Using the database we have been able to show that the introduction of AWR consultations (*n*=204) resulted in reduced antibiotic exposure (IVOST, de-escalated, stopped) in 46% of patients. Although 39% of consultations did not result in a change in antimicrobial therapy, a number of AS interventions to improve AS, patient safety and patient care, were actioned during these consultations. The most commonly recorded interventions were ongoing infection management advice, antimicrobial course length confirmation/advice and patient counselling and monitoring advice. Using the AWR database we were also able to calculate the total proportion of IV antimicrobial therapy prescribed pre (83%) and post (54%) consultation highlighting improved IV to oral switch (IVOST).

**Conclusions:**

Development of the database has supported more timely data collection, measurement and feedback of the impact of the AWR service on AS and patient-centred care. This is important in supporting a continuous QI approach to AS and patient-centred care in the acute hospital setting.
